# Identification of Potential Key Genes in Prostate Cancer with Gene Expression, Pivotal Pathways and Regulatory Networks Analysis Using Integrated Bioinformatics Methods

**DOI:** 10.3390/genes13040655

**Published:** 2022-04-08

**Authors:** Mohd Mabood Khan, Mohammad Taleb Mohsen, Md. Zubbair Malik, Sali Abubaker Bagabir, Mustfa F. Alkhanani, Shafiul Haque, Mohammad Serajuddin, Mausumi Bharadwaj

**Affiliations:** 1 Division of Molecular Genetics & Biochemistry, National Institute of Cancer Prevention & Research (ICMR-NICPR), I-7, Sector-39, Noida 201301, India; mabood5333@gmail.com (M.M.K.); abutaleb19900@gmail.com (M.T.M.); 2 Department of Zoology, University of Lucknow, Lucknow 226007, India; serajuddin2164@redifmail.com; 3 Department of Biosciences, Jamia Millia Islamia (A Central University), New Delhi 110025, India; 4 School of Computational and Integrative Sciences, Jawaharlal Nehru University, New Delhi 110067, India; zubbairmalik@jnu.ac.in; 5 Department of Biotechnology, Jamia Hamdard University, New Delhi 110062, India; 6 Department of Medical Laboratory Technology, Faculty of Applied Medical Sciences, Jazan University, Jazan 45142, Saudi Arabia; sbagabir@jazanu.edu.sa; 7 Emergency Medical Service Department, College of Applied Sciences, AlMaarefa University, Riyadh 11597, Saudi Arabia; mkhanani@mcst.edu.sa; 8 Research and Scientific Studies Unit, College of Nursing and Allied Health Sciences, Jazan University, Jazan 45142, Saudi Arabia; shafiul.haque@hotmail.com

**Keywords:** prostate cancer, benign prostate hyperplasia, differentially expressed genes, key genes, bioinformatics

## Abstract

Prostate cancer (PCa) is the most prevalent cancer (20%) in males and is accountable for a fifth (6.8%) cancer-related deaths in males globally. Smoking, obesity, race/ethnicity, diet, age, chemicals and radiation exposure, sexually transmitted diseases, etc. are among the most common risk factors for PCa. However, the basic change at the molecular level is the manifested confirmation of PCa. Thus, this study aims to evaluate the molecular signature for PCa in comparison to benign prostatic hyperplasia (BPH). Additionally, representation of differentially expressed genes (DEGs) are conducted with the help of some bioinformatics tools like DAVID, STRING, GEPIA, Cytoscape. The gene expression profile for the four data sets GSE55945, GSE104749, GSE46602, and GSE32571 was downloaded from NCBI, Gene Expression Omnibus (GEO). For the extracted DEGs, different types of analysis including functional and pathway enrichment analysis, protein–protein interaction (PPI) network construction, survival analysis and transcription factor (TF) prediction were conducted. We obtained 633 most significant upregulated genes and 1219 downregulated genes, and a sum total of 1852 DEGs were found from all four datasets after assessment. The key genes, including *EGFR*, *MYC*, *VEGFA*, and *PTEN*, are targeted by TF such as AR, Sp1, TP53, NF-KB1, STAT3, RELA. Moreover, miR-21-5p also found significantly associated with all the four key genes. Further, The Cancer Genome Atlas data (TCGA) independent database was used for validation of key genes *EGFR*, *MYC*, *VEGFA*, PTEN expression in prostate adenocarcinoma. All four key genes were found to be significantly correlated with overall survival in PCa. Therefore, the therapeutic target may be determined by the information of these key gene’s findings for the diagnosis, prognosis and treatment of PCa.

## 1. Introduction

Prostate cancer (PCa) is the most prevalent cancer (20%) in males and accountable for a fifth (6.8%) cancer-related deaths in males globally [1]. According to the World Cancer Survey Statistics (GLOBOCAN), the number of newly diagnosed cases of PCa was ~1.41 million in 2020, with ~375 thousand new deaths [2,3]. By 2040, the global PCa burden is expected to increase to 2.43 million new cases and 740 thousand new deaths due to population growth and ageing [2,3]. On the other hand, PCa incidence has been steadily increasing in India. According to India’s population-based cancer registries, PCa is the second most common cause of cancer in men living in metropolitan areas [4,5].

Smoking, obesity, race/ethnicity, diet, age, chemicals and radiation exposure, sexually transmitted diseases, etc., are among the most common risk factors for prostate cancer [6]. However, the basic change at the molecular level is the manifested confirmation of PCa. Although prostate specific antigen (PSA) level is the most frequently used screening tool for prostate cancer detection, it doesn’t stand as an absolute method to predict disease malignancy. Furthermore, the use of genetic profiling may provide additional benefits for early PCa detection [7,8,9,10]. Prostate cancer may be rectified at an early stage of cancer by surgery or radiation therapy, but patients with advanced or metastatic disease may have no curative therapeutic options [11,12]. Consequently, there is an urgent need for treatments capable of preventing or combating PCa.

Currently, gene expression data mining and bioinformatics microarray analysis are widely used to find key genes for disease severity, pathogenesis, complexity, recognition of suitable biomarkers, miRNA targets, transcription factor (TF) recognition and drug targets. The gene expression is a molecular signature used as a diagnostic and prognostic marker in cancer research. Gene expression profiling reveals several differentially expressed genes (DEGs) in patient samples, but many genes cannot be related by the prevailing methods. Protein-protein interaction (PPI) network research plays a great role in understanding the molecular function; and any malfunctioning pathway linked to the disease network may result from changes in the PPI network locality. PPI gives elaboration on protein function, molecular magnitude, gene ontology (GO), disease regulator genes, miRNA and novel drug targets.

Previous PCa meta-analysis studies have revealed mostly one type of molecular markers, like genetic molecular markers by Zhao et al., (2014) who reported *Ki-67*, *Bcl-2*, *CD147*, *COX-2*, *ALDH1A1* and *FVIII* genes [13]. Or miRNAs as reported by Song et al., (2017) of up/down regulated miRNAs like miR-18a, miR-34a, miR-129, miR-145, etc. [14] However, limited studies have reported different types of biomarkers in the same study [15]. This study aims to evaluate the molecular-signature for PCa in comparison to benign prostatic hyperplasia (BPH). Additionally, in this study, identification and classification of DEGs were conducted with the help of some bioinformatics tools like DAVID (to know about the functional annotation of a gene), STRING (to find out PPI network functional enrichment analysis), GEPIA (to find differential gene expression analysis and profiling plotting), and Cytoscape (to make an interaction map). Furthermore, to find key genes associated with pathogenesis, the degree of network interaction as well as the module of the most related gene; are used to discover hub genes that may work as key targets for treatment.

## 2. Materials and Methods

### 2.1. Microarray Data Extraction

The National Centre for Biotechnology Information (NCBI)-Gene Expression Omnibus (GEO) (https://www.ncbi.nlm.nih.gov/geo/, accessed on 11 March 2022) [16] is a global public domain for gene expression database from which we have retrieved the four data sets (GSE55945, GSE104749, GSE46602, GSE32571) belonging to the prostate tumor (*n* = 112) and the BPH tissue (*n* = 65). Only datasets that met the following inclusion and exclusion criteria for PCa datasets were incorporated in this study. First, inclusion criteria take in (i) datasets with expression data for PCa tissue and BPH tissue samples; (ii) datasets with at least four samples’ gene expression data (both PCa tumor and BPH) and (iii) homo sapiens datasets. Table 1 summarizes all of the information regarding the datasets that were chosen. The matrix files of GSE55945, GSE104749, GSE46602, GSE32571 were obtained by GEO2R, and the raw data of the datasets were downloaded by the GEO query package of the R language.

### 2.2. Differentially Expressed Genes Identification

**Figure 1:**
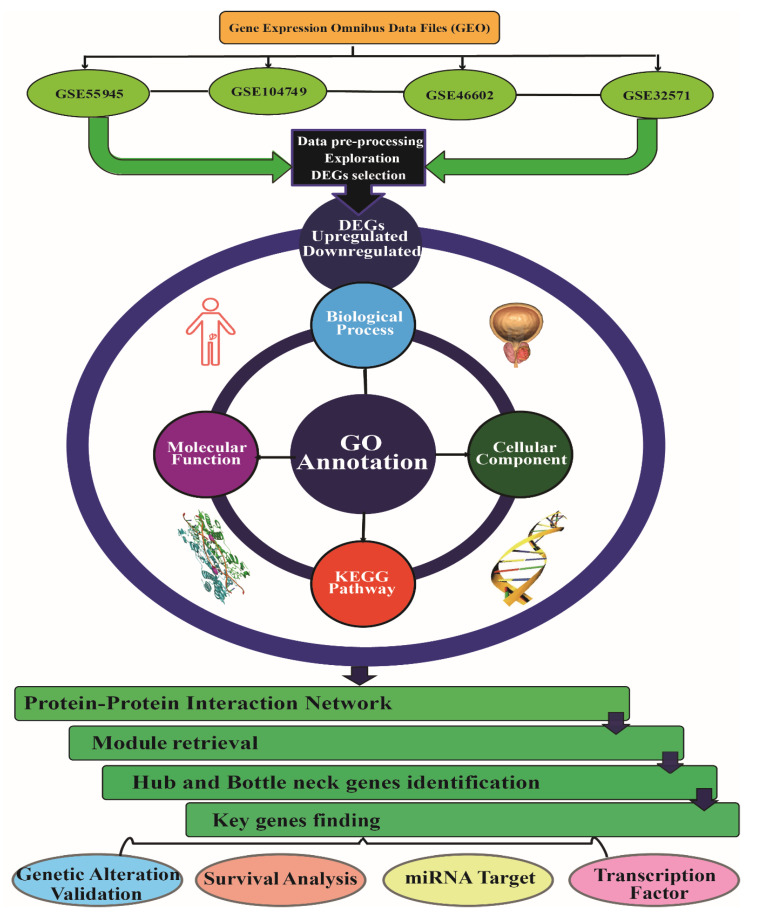
Flowchart of methodology containing sorting of GEO datafile, selection of DEGs, GO annotation analysis, and identification of key genes. DEGs (differentially expressed genes), GEO (gene expression omnibus), GO (gene ontology).

The study was carried out in accordance with the flowchart shown in Figure 1. The differentially expressed genes (DEGs) in GSE55945, GSE104749, GSE46602, and GSE32571 were obtained with GEO2R analysis of the GEO database. In brief, the GEO2R tool of the GEO database was used to extract the matrix files for GSE55945, GSE104749, GSE46602, and GSE32571 from their respective GSE databases. There were 13 prostate tumor and 8 BPH tissue samples for GSE55945, 4 prostate tumor and 4 BPH tissue samples for GSE104749, 36 prostate tumor and 14 BPH tissue samples for GSE46602, 59 prostate tumor and 39 BPH tissue samples for GSE32571. These four datasets were chosen for further GEO2R investigation. Moreover, mRNAs having an adjusted *p*-value < 0.05 and [log2FC] > +1, [log2FC] < −1 were chosen as significant DEGs. The DEGs were visualised in a volcano plot using the R package “ggplot2” (https://cran.r-project.org/web/packages/ggplot2/ accessed on 18 February 2022). The BRCW computing website (http://jura.wi.mit.edu/bioc/tools/compare.php, accessed on 18 March 2022) was used to choose unique DEGs that were shared by at least two gene expression profile datasets. As a result, we were able to be more precise in our DEG selection, and the possibility of biassed data compilation was reduced to a negligible level. The Venn Diagram was used to visualise the upregulated, downregulated, and unique DEGs identified from four datasets.

### 2.3. Gene Ontology and Pathway Enrichment Analysis

The DEGs functional interpretation was evaluated and visualized in web resource such as DAVID (DAVID; version 6.7, http://david.abcc.ncifcrf.gov, accessed on 18 February 2022) [24] as per the molecular function, biological process and cellular component of DEGs. For the metabolic pathway enrichment study, the Kyoto Encyclopedia of Genes and Genomes (KEGG) pathways (http://www.genome.jp/kegg, accessed on 18 February 2022) [25] tool was used. The adjusted *p*-value < 0.05 cutoff score was taken into consideration for obtaining significant expressed genes.

### 2.4. Protein-Protein Network Screening (PPI), Key Genes Identification and Module Network Construction

The online program STRING (http://www.string-db.org/, accessed on 18 February 2022) was used to extract interconnected genes to create a network of PPI [26]. To visualize PPI, the tool Cytoscape version 3.8.2 (http://www.cytoscape.org/, accessed on 18 February 2022) [27] was introduced. To identify significant genes in the subnetwork, a Cytoscape plugin Molecular Complex Detection (MCODE) was implemented with the parameters K-score (2), degree cutoff score (2), node cutoff score (0.2) and 100 maximum depths. To find the most intersected key genes and modules, the Cytohubba plugin tool (http://hub.iis.sinica.edu.tw/cytohubba/, accessed on 18 February 2022) [28] was used in Cytoscape and the PPI-MCODE modules were also merged. Multiple topological characteristics such as Maximal Clique Centrality, Density of Maximum Neighborhood, Betweenness Centrality, Closeness Centrality, Degree, Stress and Bottleneck were also added to the network for identification of key genes and modules.

### 2.5. Genetic Alteration and Validation of Key Genes Expression Paradigm

As per the manual, examination of genetic alteration for key genes was performed by the cBioportal for Cancer Genomics [29]. The oncoplot for key genes was created by the cBioPortal for Cancer Genomics. Further, the GEPIA (Gene Expression Profiling Interactive Analysis) online software (http://gepia.cancer-pku.cn, accessed on 18 February 2022) was used for investigation of key genes expression in PCa. The verification of key gene expression was conducted by GEPIA between 492 PCa and 152 non-cancer tissues. GEPIA has synergetic and adjustable features such as analysis of differential expression, analysis of correlation and analysis of patient survival and can provide rapid results from The Cancer Genome Atlas (TCGA) data [30]. The values greater than the transcripts median were classified as increased expressions, and the values lower than the transcripts median were classified as decreased expressions.

### 2.6. Survival Analysis of Key Genes

The online web UALCAN based on the TCGA database was used for the survival analysis of key genes expression in PCa [31]. The transcript per million (TPM) enrichment analysis was used for classification of PCa patients’ expression into high and medium/low expression. The Kaplan-Meier (KM) survival analysis (*p* < 0.05) was used for the evaluation of key genes prognostic value along with Gleason Score.

### 2.7. miRNA and Transcription Factor Associated Network with Key Genes

Multiple experimentally verified online miRNA network software’s are available to estimate the miRNA interaction with genes. The miRNA selection was done through Enrichr (https://maayanlab.cloud/Enrichr/, accessed on 18 February 2022) [32] and the TRRUST online database (https://www.grnpedia.org/trrust/, accessed on 18 February 2022) [33] was used to identify transcription factors. Selection of putative target miRNA and transcription factor of key genes was done on the basis of a selected online tool. Additionally, the TransmiR v2.0 database (http://www.cuilab.cn/transmir, accessed on 18 February 2022) [34] was used to see the association between transcription factors and miRNAs. Further validation of miRNA along with TF for key genes was done by the online public database miRNet (https://www.mirnet.ca/, accessed on 18 February 2022) visual interaction platform [35]. The Cytoscape was used for the formation of an integrative network of key genes, miRNAs, and transcription factors on the basis of source and target association.

## 3. Result

### 3.1. Deferentially Expressed Genes Discovery

**Figure 2:**
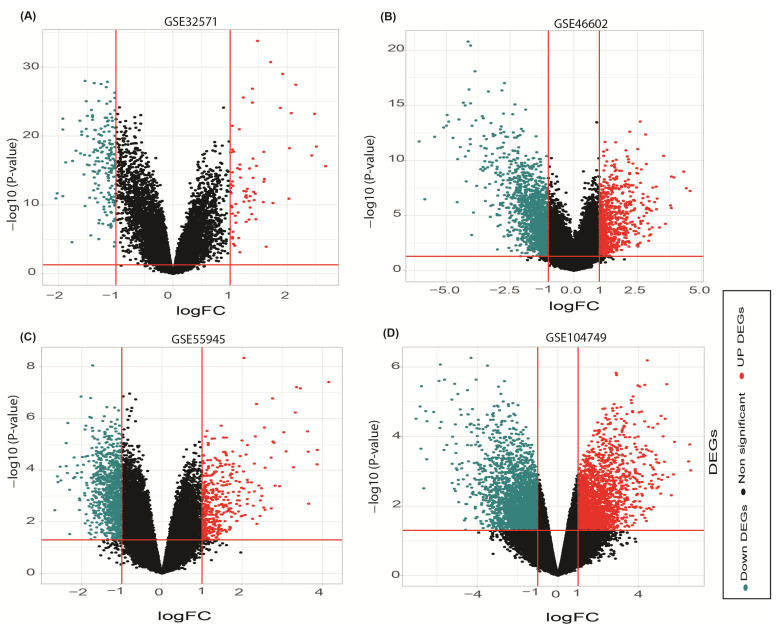
(**A**–**D**) Volcano plots showing DEGs between prostate cancer and benign prostate hyperplasia patients in GSE32571, GSE46602, GSE55945 and GSE104749 datafiles. The log2FC value > 1, log2FC value < −1 and *p* < 0.05 are the cutoff value for significant upregulated (red color), non-significant (black color) and downregulated (cyan color) DEGs.

A total number of 164,526 annotated transcripts were obtained from the included GSEs evaluated in the study, 132 upregulated and 387 downregulated DEGs were selected in the GSE55945 data files on the basis of selection criteria (adjusted *p*-value < 0.05 and [log2FC] > +1, [log2FC] < −1) as compared between prostate cancer and BPH patients. Subsequently, 98 upregulated and 122 downregulated DEGs were identified in the GSE104749 data files, 501 upregulated and 892 downregulated DEGs in the GSE46602 data files, 60 upregulated and 166 downregulated DEGs in the GSE32571 data files from the same criteria selection. The most significant upregulated and downregulated genes for the GSE55945, GSE46602, GSE104749, GSE32571 data set are shown in the Supplementary file S1. The Volcano plots showed upregulated and downregulated genes for all datasets by implementing upper and lower limit criteria [logFC] > +1, [logFC] < −1 (Figure 2). Pre raw value and post normalized value is indicated by all four-dataset box plots (Supplementary file S2). A comparison of the complete gene and top 100 genes expression profile of prostate cancer versus benign prostate hyperplasia (BPH) was further demonstrated in a graded manner by heatmap construction (Supplementary file S3).

### 3.2. DEGs Functional Annotation and KEGG Pathway Analysis

**Figure 3:**
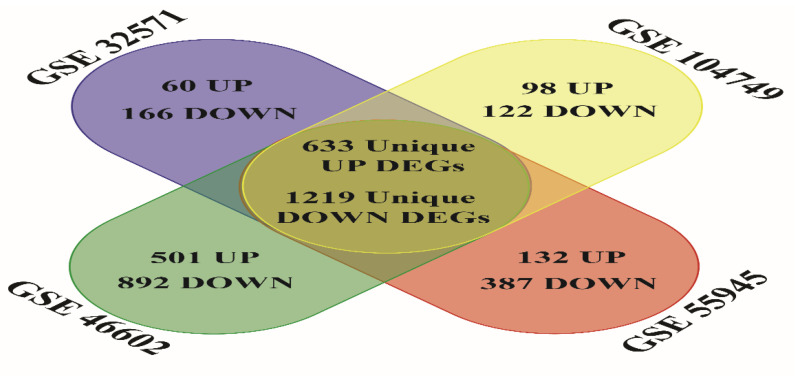
A plot depicting the upregulated-downregulated & unique DEGs discovered in four datafiles GSE32571, GSE104749, GSE46602, GSE55945. Statistically significant DEGs were characterized by the cut-off criterion of log2FC value > 1, log2FC value < −1 and *p* < 0.05.

**Figure 4:**
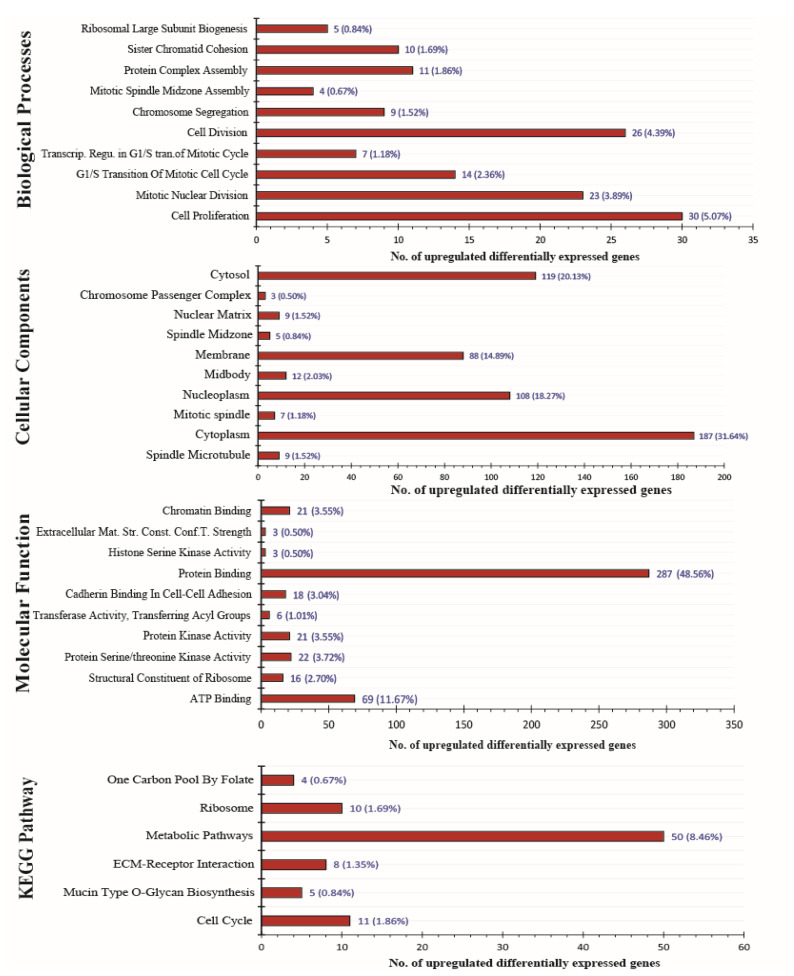
Gene ontology functional classification and pathway analysis of significant differentially expressed genes from various datafiles. Bar-graph of top most significant upregulated genes representing biological process, cellular component, molecular function, KEGG pathway analysis. KEGG (Kyoto Encyclopedia of Genes and Genomes).

**Figure 5:**
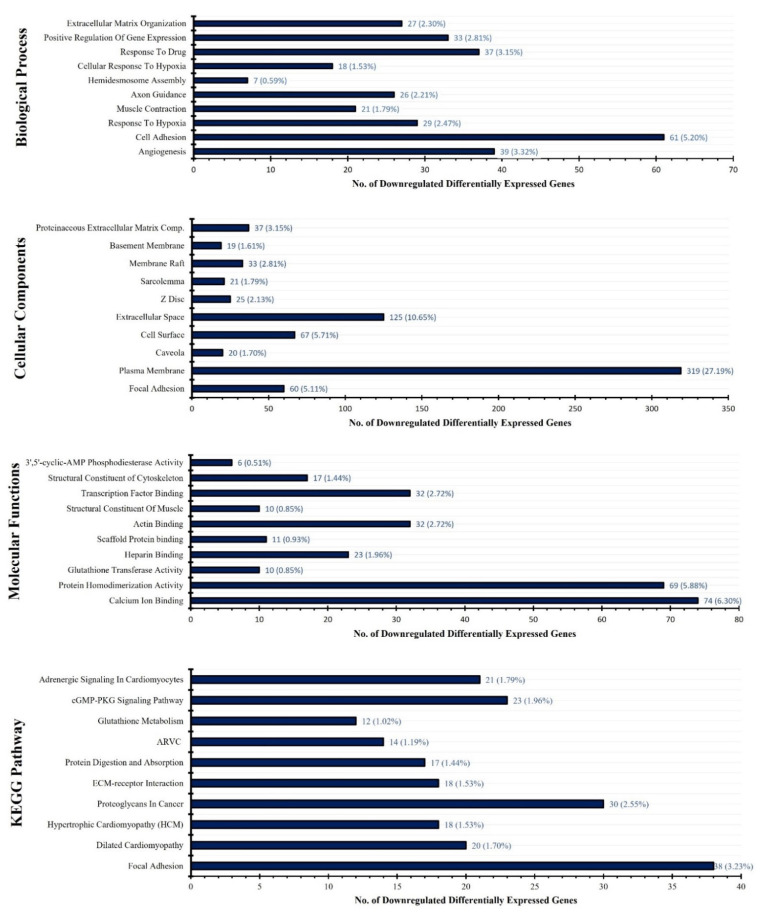
Gene ontology functional classification and pathway analysis of significant differentially expressed genes from various datafiles. Bar-graph of top most significant downregulated genes representing biological process, cellular component, molecular function, KEGG pathway analysis. KEGG (Kyoto Encyclopedia of Genes and Genomes).

To classify unique upregulated and downregulated of all four data sets GSE55945, GSE104749, GSE46602 and GSE32571 the bioinformatics and research computing online tool (http://barc.wi.mit.edu/tools/, accessed on 18 March 2022) was used. We obtained 633 most significant unique upregulated genes and 1219 unique downregulated genes from all four datasets after assessment (Figure 3). In order to evaluate the gene-ontology functional analysis of selected DEGs for biological process (BP), cellular process (CC), molecular function (MF) and KEGG pathway significance, DAVID online software was used. The most significant biological process for upregulated genes involved in cell proliferation (FDR:0.003528782), mitotic nuclear division (FDR:0.005441812), G1/S transition of mitotic cell cycle (FDR:0.007227173) and most significant biological process for downregulated genes involved in angiogenesis (FDR:7.53 × 10^−6^), cell adhesion (FDR:7.53 × 10^−6^), response to hypoxia (FDR:0.001023707). The most significant upregulated genes were available in the portion of the spindle microtubule (FDR:0.012514875), cytoplasm (FDR:0.093997223), mitotic spindle (FDR:0.093997223), and the most significant downregulated genes were available in the area of focal adhesion (FDR:2.39 × 10^−9^), plasma membrane (FDR:5.86 × 10^−8^), caveola region (FDR:2.95 × 10^−7^) under the cellular component category. Molecular function enrichment assessment for most significant upregulated genes included ATP binding activity (FDR:0.149334827), structural constituent of ribosome activity (FDR:0.704002703), protein serine/threonine kinase activity (FDR:0.704002703) and for most significant downregulated genes included calcium ion binding (FDR:0.002557467), protein homodimerization activity (FDR:0.042898325), glutathione transferase activity (FDR:0.042898325). The most significant upregulated genes under KEGG pathway analysis are involved in Cell cycle signaling (FDR:0.74589722), Mucin type O-Glycan biosynthesis pathway (FDR:0.74589722), ECM-receptor interaction pathway (FDR:0.74589722) and in the case of the most significant downregulated genes under KEGG pathway analysis, they are involved in Focal adhesion (FDR:2.42 × 10^−7^), Dilated cardiomyopathy (FDR:4.04 × 10^−5^), Hypertrophic cardiomyopathy (HCM) signaling pathway (FDR:1.94 × 10^−4^) (Figure 4 and Figure 5, Supplementary files S4–S11).

### 3.3. Protein-Protein (PPI) Network and Module Analysis

**Figure 6:**
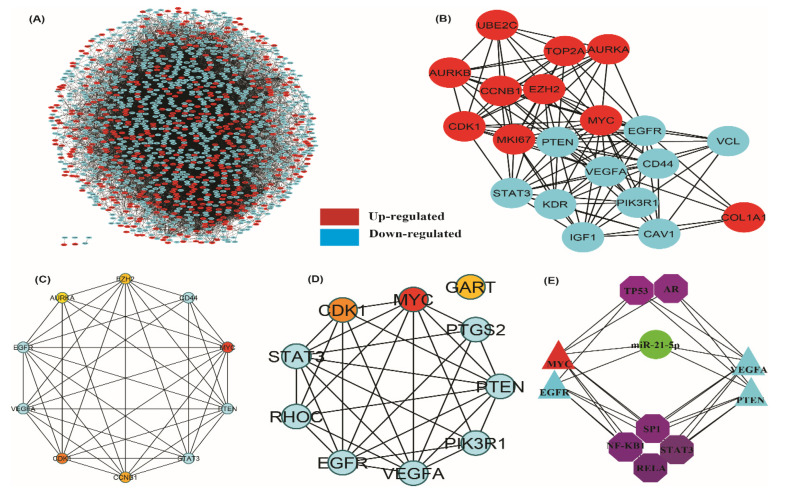
(**A**) Most significant upregulated and downregulated genes PPI network map. (upregulated genes represented by red colour and downregulated genes represented by blue colour) (**B**) Top 10 most connected upregulated and downregulated genes according to their highest degree score associated with prostate cancer. (upregulated genes represented by red colour and downregulated genes represented by blue colour) (**C**) Top 10 hub significant genes extracted by PPI Cyto-hubba network & (**D**) Top 10 bottleneck significant genes excavated from PPI Cyto-hubba network. Higher score is represented by colour intensity in hub and bottleneck genes both. (upregulated genes represented by red colour and downregulated genes represented by blue colour) (**E**) Deep network extraction showing the key genes, TF and miRNA interaction with each other. (upregulated genes represented by red colour, downregulated genes represented by blue colour, miRNA represented by green colour & TFs represented by violet colour).

**Figure 7:**
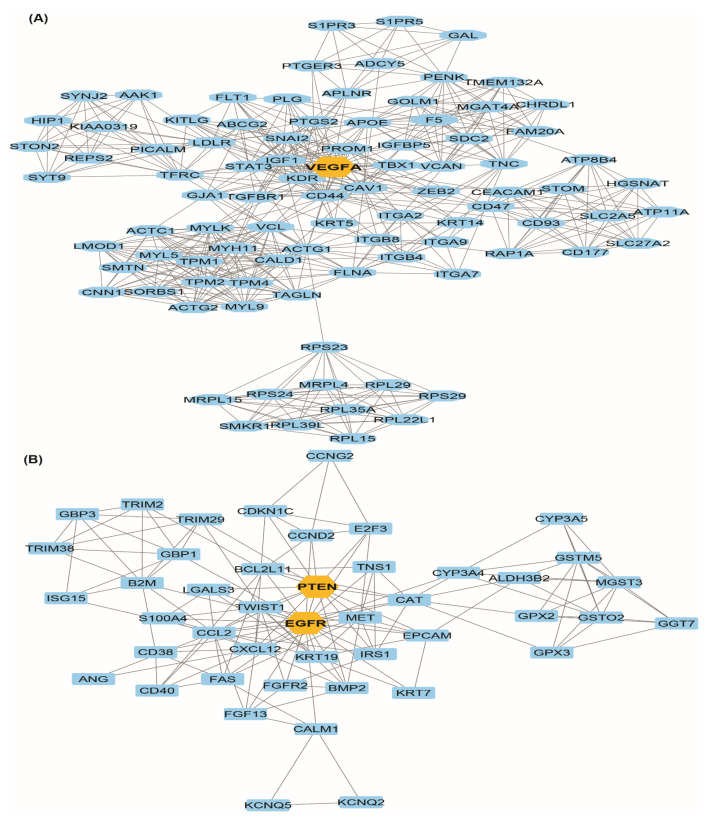
Identification of modules from PPI network. (**A**) Most significant module-A & (**B**) modules-B in the MCODE-PPI network analysis. DEGs is represented by nodes and interaction between them represented by edges.

In the Cytoscape platform for up regulated and downregulated genes, we found 1572 nodes, 11,279 edges and 13.8 mean node degree after topological property analysis of the PPI network (Figure 6A). After topological enrichment, the most connected upregulated genes *MYC*, *CDK1, CCNB1* etc. and the most connected downregulated genes *EGFR*, *VEGFA, STAT3* etc. were categorized according to their highest degree count associated with prostate cancer (Figure 6B and Table 2). The characterization of this newly synthesized PPI with MCODE score ≥ 3 and nodes ≥ 3 in the retrieval of two modules. Module A had 92 nodes, 580 edges with an MCODE score of 12.747 and module B had 45 nodes, 153 edges with an MCODE score of 6.955 (Figure 7A,B).

### 3.4. Hub-Bottle Neck Genes and Key Genes Identification

A Cytoscape plug-in, Cytohubba was applied to the established PPI network together with the decreasing score of the Degree algorithm to deduce the top most hub genes. The shortest path, centrality algorithm along with bottle neck score were used in Cytohubba software to discover the top most bottle neck genes. For further investigation, the top 10 hub genes (Figure 6C) and the top 10 bottle neck genes were selected (Figure 6D). After study, *EGFR*, *MYC*, *VEGFA*, *PTEN*, *STAT3*, *CDK1*, a total of six were found common in both hub and bottleneck genes, along with the most connected genes were *EGFR* and *VEGFA*. There were four unique hub genes including *CCNB1*, *EZH2*, *AURKA* and *CD44*, and four unique bottleneck genes, including *PIK3R1*, *GART*, *PITGS2* and *RHOC* (Supplementary file S12). After taking certain topological algorithms such as betweenness centrality, closeness Centrality, degree score, stress, bottleneck score, we identified four key regulator genes including *EGFR*, *MYC, VEGFA, PTEN*. According to our findings, common TFs such AR, Sp1, TP53, NF-KB1, STAT3, and RELA are targeted by all identified four key genes. Also, miR-21-5p has a strong connection to each of the four key genes investigated in this study (Figure 6E). The genes follow more than one criterion such as (or including?) *STAT3*, *CAT*, *VCL*, *EZH2*, *CD44*, *CAV1*; unique genes *C11or f96*, *TMEM106C*, *DMKN*, *PRELID2*, *UBXN10*, *ZNF613*, *CCNB1*, *AURKA*, *GART*, *PTGS2*, *RHOC*, *PIK3R1* were revealed after integrated analysis (Supplementary file S12).

### 3.5. Genetic Alteration and Validation of Four Key Genes Expression Paradigm

**Figure 8:**
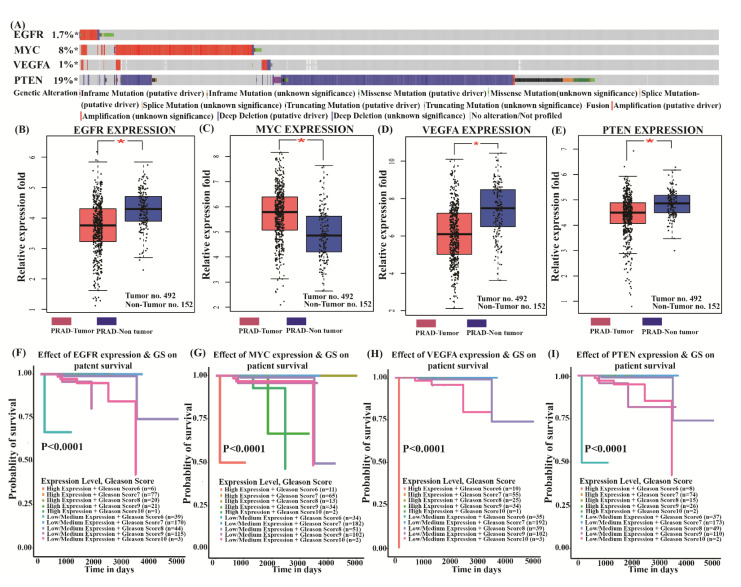
Genetic alteration, validation and survival analysis of four key genes expression paradigm related to PCa using TCGA data. (**A**) The oncoplot of four key genes. (**B**–**E**) The validation of four key genes expression paradigm in PRAD (*n* = 492) tumor and non-tumor (*n* = 152). (**F**–**I**) Survival analysis of four key genes associated with PCa using Kaplan Meier method, * Significant *p*-value < 0.05.

After statistical analysis by cBioPortal for Cancer Genomics, we observed that 1766 (26%) of the 6875 prostate cancer patients documented genetic alteration in the four key genes. Deletion and amplification were the utmost prevalent genetic variations. (Figure 8A). Further, the GEPIA tool (http://gepia.cancer-pku.cn, accessed on 18 February 2022) was used for the evaluation of key genes *EGFR*, *MYC*, *VEGFA*, *PTEN* expression in The Cancer Genome Atlas data. As per the GEO dataset findings, the key genes *EGFR*, *VEGFA*, *PTEN* expression level in prostate adenocarcinoma (PRAD) was significantly lower (*p* < 0.05) compared to non-tumor prostate tissue and the *MYC* expression level was significantly higher (*p* < 0.05) in prostate adenocarcinoma (PRAD) compared to non-tumor prostate tissue verified by the TCGA database (Figure 8B–E).

### 3.6. Survival Analysis of Key Genes

The TCGA based UALCAN transcriptomic cancer data was used for the survival assessment of PCa patients and gene expression analysis. The four key regulator genes expression were analyzed in the UALCAN database using the Kaplan Meier method. The classification of PCa tumor tissue is based on the Gleason Score (GS) method, according to this grading system, gleason score GS ≤ 6, 3 + 4, 4 + 3, 8, 4 + 5, 5 + 4, 10 is related to Gleason Grading Group 1, 2, 3, 4, and 5 respectively [36]. It was noted that all the four key genes were found significantly correlated with overall survival in PCa. The overall survival of the low/medium expression group was observed to be significantly lower than the high expression group for *EGFR*, *VEGFA, PTEN* genes and the overall survival of the high expression group was observed to be significantly lower than the low/medium expression group for *MYC* genes in PCa patients after integrated analysis with the Gleason Score system (Figure 8F–I).

### 3.7. miRNA and Transcription Factor Associated Network with Key Genes

The miRNAs play an important role in gene expression regulation at multiple stages after RNA synthesis. miRNA up and downregulation defects are linked with prostate cancer and they have an ability to differentiate between benign and malignant tumors [37] and the disease complexity can be more readable by miRNA changes. The four key identified genes were connected to approximately 394 miRNAs (Supplementary file S13) and 233 transcription factors (Supplementary file S14), which could be responsible for controlling key genes. Further results demonstrated *EGFR*-associated 38 miRNAs, *MYC*-associated 115 miRNAs, *VEGFA*-associated 121 miRNAs, *PTEN*-associated 120 miRNAs. Moreover, TF analysis revealed the following: *EGFR*-associated 32 transcription factors, *MYC*-associated 95 transcription factors, *VEGFA*-associated 83 transcription factors and *PTEN*-associated 23 transcription factors. In addition, the AR transcription factor associated with 13111 miRNAs possible binding sites, STAT3 transcription factor associated with 72 miRNAs and RELA transcription factor associated with 227 miRNAs in PCa (Supplementary file S15).

## 4. Discussion

Prostate cancer is a widely spread one across the world among males. PCa can metastasize through the circulatory system reaching distant parts of the body [38]. The disease’s pathogenicity, magnitude detection, ambiguity, predictive and clinical biomarker scarcity are the main barriers in the path of PCa care [6,39]. As a result, the integrated PCa key regulatory genes profiling may help to achieve more successful treatment of PCa patients. This research has highlighted the DEGs related to PCa out of 112 tumor samples and 65 BPH tissues, pooled from GSE 55945, GSE 104749, GSE46602, GSE32571 datasets, with the help of bioinformatic techniques. As a result, a total number of 1866 significantly DEGs were found, in which there were 638 up and 1228 downregulated genes. In order to demonstrate the relationship between up and down DEGs with PPI network formation, two modules were found to be significantly crucial in this PPI network study. Taking this into the account, the highest ranked genes were screened by Cytohubba in terms of hub and bottle neck genes. These techniques of gene ontology (GO) and KEGG pathways were used to describe the role of DEGs. The top characterized and screened genes including *EGFR*, *MYC, VEGFA, PTEN* which are involved in prostate cancer progression, cell proliferation and division, cell cycle signaling, metabolic and signaling pathway regulation, angiogenesis, focal adhesion etc. [40,41,42,43,44] Which makes them further as molecular candidates involved in treatment. Furthermore, various hub genes and bottle neck genes linked to prostate cancer were uncovered through this study. In addition to that, identification of miRNAs and screening of the transcription factors revealed molecular markers for prostate cancer progression control.

The Epidermal Growth Factor Receptor (EGFR) is involved in many biological processes, such as proliferation, vitality, migration, progression and cell signaling [40,41]. By contrast, *EGFR* under expression has been reported with tumor progression [42]. In the Pakistani population, Hashmi and his colleagues have shown that *EGFR* under expression was linked to prostatic adenocarcinoma, suggesting it as clinical biomarker in cases with higher Gleason score, high grade and perineural association with prostate carcinoma [43]. Recent evidence has shown that *EGFR* and the related AKT pathway are effectively associated with AR phosphorylation [44], but it has been observed in PCa that an inverse relationship has been seen in terms of *EGFR* and AR protein/expression [45]. The regulators for *EGFR*, TF and microRNAs included miRNA-145 which is boosted by TF p53 (TP53) and inhibits *EGRF* expression [46,47,48]. Also, miR-199a-3p which targets *EGFR* as well as *c-Myc* [49,50].

In the light of PCa progression, *MYC* is another significant gene. *C-Myc*, *N-myc* and *L-myc* are *MYC* subtypes that encode similar MYC protein which have the same function [51,52]. The level of expression is contrasted in various tissues, such as *N-myc* upregulated in solidified tumor glioma and neuroblastoma, *c-Myc* upregulated in solidified cancer and blood-related cancer, acting as proto-oncogene as well as transcription factor while *L-myc* upregulated in lung carcinoma [53]. *C-Myc* is a critical factor in PCa progression and related to cell expansion [54,55] which goes together with our findings. Inhibition of *c-Myc* could be carried by multiple miRNAs, including miR-let-7a which also has a tumor suppression function in PCa cells through downregulating AR expression [56]. Also, miRNA-34a plays a critical role as a tumor suppressor along with p53 arbitrator activity, which has been down expressed in PCa; it lacks the capability to suppress *c-Myc* in PCa cells [57]. Also, miR-23b can play the same role as miR-34a, but other oncogenic transcription factors like NF-KB and Sp1 can activate the alternative way in *MYC*-dependent miR-23b inhibition, for cell survival and growth [58] which is shown in our TF data.

The VEGF family’s key role is to facilitate angiogenesis in malignant cancer, making it an effective therapeutic candidate for tumor disease cure. Bender and co-workers speculated that primary prostate tumors experiencing VEGFA (vascular endothelial growth factor-A) low expression [59,60]. The VEGF level could be regulated by EGFR’s action via the signaling network of mitogen-activated protein kinase (MAPK) as well as phosphoinositide 3-kinase (PI3K) [61] which has been observed in our finding for *EGFR*. In this study, the obtained final TFs and microRNAs have a direct connection with *VEGFA*, like AR, TP53, SP1, miR-299-3p [62,63] which have been found and reported in our supplementary file.

PTEN (Phosphatase and Tensin Homolog) belongs to the phosphatase group that regulates the signaling pathway of PI3K as well as AKT [64]; PTEN has tumor suppressor activity and is generally found idle connected to PCa [65]. Downregulated *PTEN* expression in our findings goes in line with the previously reported results in Iranian PCa patients [66]. PTEN plays a reverse role in the signaling effect of PI3K/AKT and dephosphorylates PIP3 [64,67]. Also, PTEN has the capability to inhibit AR in a clinical manner through AR nuclear translocation blocking as well as depletion of AR protein, and this matches AR over expression as per our findings [68,69,70]. Regulation of *PTEN* was targeted by miRNA-let-7b and miR-548 and down expression of miR-let-7b (tumor suppressor) was detected in our findings, but *PTEN* was not correlated with miR-548 in terms of expression [62]. Another finding indicated that miR-21 and miR-181b-1 both repressed *PTEN* as well as *CYLD*, which were subsequently turned on by STAT3 in the signaling [71].

In addition, the examined PPI network was strongly correlated with the rest of the hub and bottleneck genes which are essential genes in the network pathway operations. In this study, DEGs were obtained through a comparison between PCa and BPH samples. After that, the PPI network was established and chosen for further analysis. The significance of this study lies in detecting and revealing unique key hub genes including *EGFR*, *MYC*, *VEGFA*, *PTEN* from different sources collectively and their regulation by common transcription factors. Besides, Androgen receptor (AR), Sp1, TP53, NF-KB1, STAT3, RELA and signature microRNAs such as miR-21-5p, miR-125a-5p, miR145-5p and miR-155-5p have been reported jointly in the same study for the first time in PCa tissue samples as tumor markers and clinical targets.

## 5. Conclusions

In comparison to BPH, our bioinformatics combined enrichment analysis revealed that key genes *EGFR*, *MYC*, *VEGFA*, and *PTEN* were identified as potent molecular biomarkers of PCa from gene expression profiling. We found that all four key genes are targeted by common transcription factors such as AR, Sp1, TP53, NF-KB1, STAT3 and RELA. Moreover, MYC as a transcription factor has a target for TP53 and shares a target with it as well. MYC shares a target with other transcription factors such as NF-KB1, STAT3 and RELA. Additionally, our analysis determined that miR-21-5p was significantly associated with all four key genes while miR-125a-5p and miR145-5p were significantly associated with *EGFR*, *MYC*, *VEGFA*, but not with *PTEN*; and miR-155-5p was significantly associated with *EGFR*, *MYC*, *PTEN*. Furthermore, we found that miR-21 is also connected with AR, STAT3 transcription factor and miR-155 is connected with AR. The clinical therapeutic target of PCa can be determined by the information in these findings as well as by giving clinical insight clues for the development of new novel PCa therapies. However, this study has a limitation of absent confirmatory experimental validation but provides a new door for future study.

## Figures and Tables

**Table 1:** Information about the included GSEs in this study.

Sample (Accession No.)	Prostate Cancer	Benign Prostate Hyperplasia	Organism	Sample Type	Platform	Reference	Included/ Excluded
GSE55945	13	8	Homo sapiens	Radical prostatectomy tissue	Affymetrix GPL570	[17]	Included
GSE104749	4	4	Homo sapiens	Fine-Needle Aspiration tissue	Affymetrix GPL570	[18]	Included
GSE46602	36	14	Homo sapiens	Laser micro dissected tissue	Affymetrix GPL570	[19]	Included
GSE32571	59	39	Homo sapiens	Fresh frozen tissue	Illumina GPL6947	[20]	Included
GSE142288	48	Nil	Homo sapiens	Tissue	Agilent GPL13264	[21]	Excluded
GSE155792	1	Nil	Homo sapiens	Tissue	Agilent GPL28148	NA	Excluded
GSE113153	10	Nil	Homo sapiens	Tissue	GPL21825	[22]	Excluded
GSE134160	164	Nil	Homo sapiens	Fresh frozen tissue	Agilent GPL26898	[23]	Excluded

**Table 2:** Top 10 highly connected up and downregulated gene.

Status	Gene Symbol	Degree	Status	Gene Symbol	Degree
Upregulated	*MYC*	166	Downregulated	*EGFR*	190
	*CDK1*	111		*VEGFA*	178
	*CCNB1*	97		*STAT3*	117
	*EZH2*	95		*CD44*	113
	*AURKA*	89		*PTEN*	110
	*UBE2C*	82		*VCL*	86
	*AURKB*	80		*IGF1*	84
	*COL1A1*	76		*CAV1*	82
	*MKI67*	76		*KDR*	79
	*TOP2A*	75		*PIK3R1*	72

## Data Availability

The data used in the current study available from the corresponding author on reasonable request.

## Supplementary Materials

The following are available online at https://www.mdpi.com/article/10.3390/genes13040655/s1, Supplementary file S1: List of the most significant upregulated and downregulated genes for the GSE55945, GSE46602, GSE104749, GSE32571 dataset. Supplementary file S2: GEO dataset processing. Boxplots show the expression intensity of each sample for the dataset GSE55945, GSE46602, GSE104749, GSE32571 before (upper panel) and after (lower panel) normalization. Supplementary file S3: The heatmap of gene expression depicts the expression levels of several genes that have undergone considerable up- or down-regulation. Upregulation (red) and down-regulation (green) are indicated by the colour. (For interpretation of the references to colour in this figure legend, the reader is referred to the web version of this article). Supplementary files S4–S7: Significant upregulated differentially expressed genes involved in GO Biological Process, Cellular Component, Molecular Function, KEGG Pathway. Supplementary files S8–S11: Significant downregulated differentially expressed genes involved in GO Biological Process, Cellular Component, Molecular Function, KEGG Pathway. Supplementary file S12: List of the key genes, hub genes, bottleneck genes, hub & bottleneck genes, unique genes, genes with more than one criterion. Supplementary file S13: List of the key genes connected to miRNAs. Supplementary file S14: List of the key genes connected to transcription factor. Supplementary file S15: List of the transcription factor connected to miRNAs.
