# Supplementary figures and images for: Identification of Potential Key Genes in Prostate Cancer with Gene Expression, Pivotal Pathways and Regulatory Networks Analysis Using Integrated Bioinformatics Methods
Source: Genes (Basel). 2022 Apr 8;13(4):655. doi: 10.3390/genes13040655 (PMC9030534; doi:10.3390/genes13040655)

# Raw Value

GSE32571

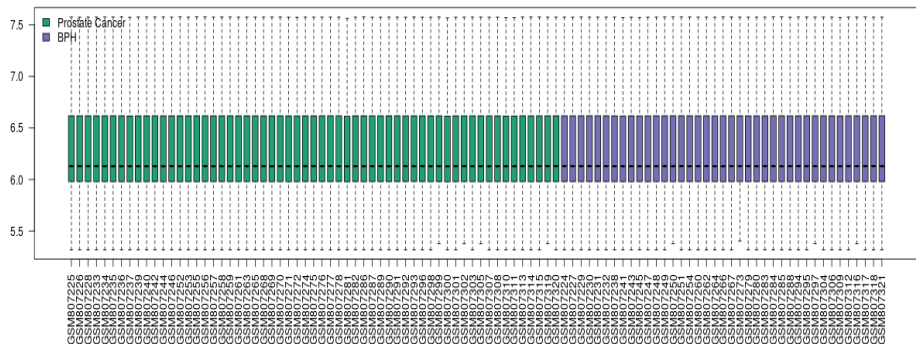

GSE46602

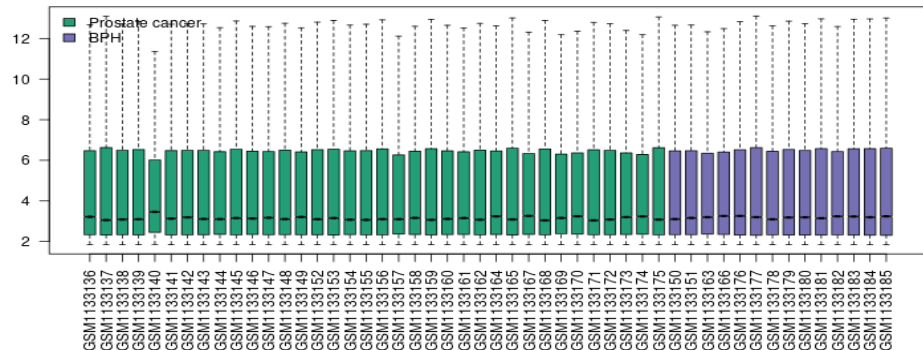

GSE55945

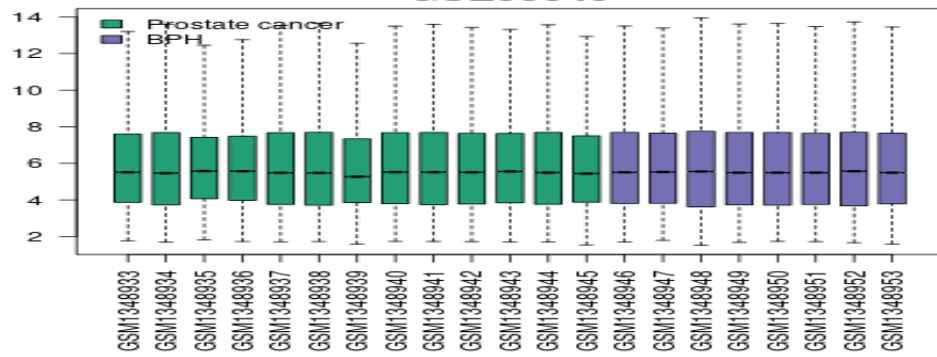

GSE104749

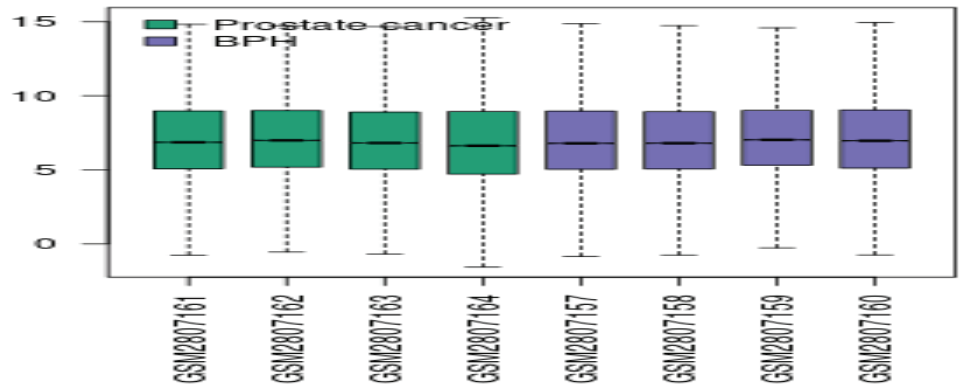

GSE32571

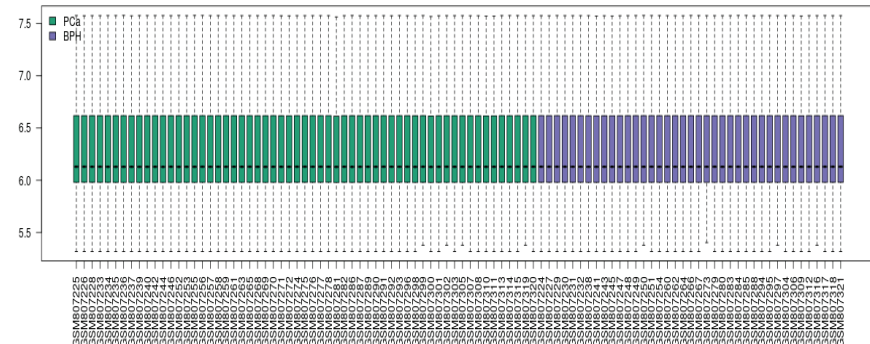

GSE46602

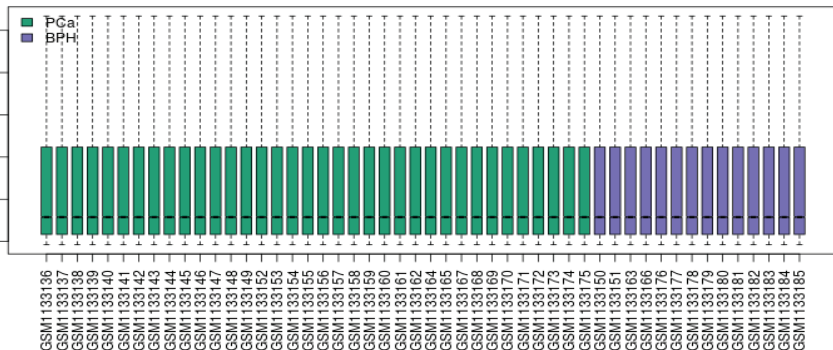

GSE55945

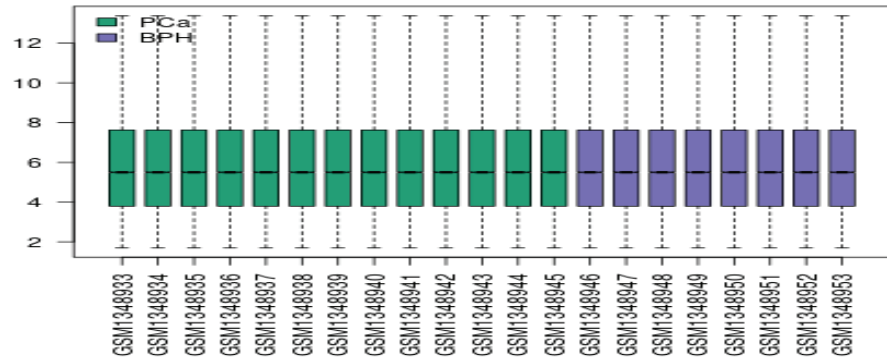

GSE104749

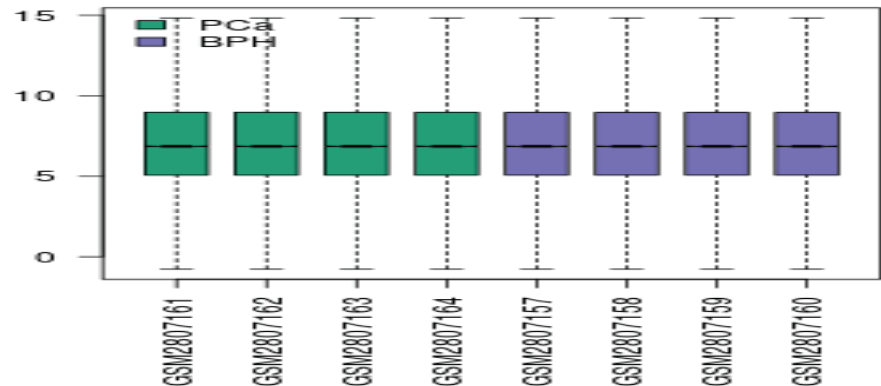

# Normalised Value

Supplement: Supplementary file 1 [file genes-13-00655-s001.zip › Supplimentary file/Supplementary file S 2.pdf]

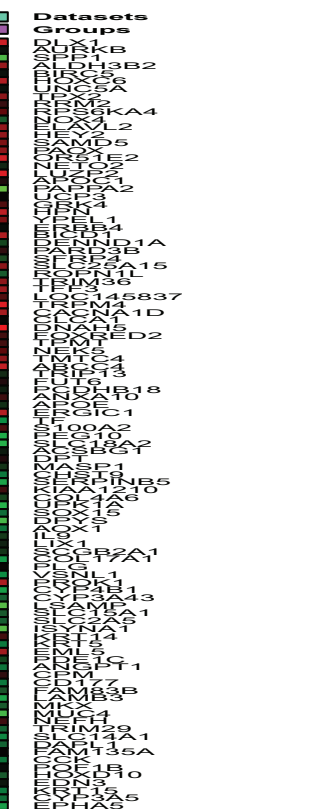

Supplement: Supplementary file 1 [file genes-13-00655-s001.zip › Supplimentary file/Supplementary file S 3.pdf]
